# Renal transplantation in a HIV positive patient

**DOI:** 10.4103/0971-4065.57110

**Published:** 2009-07

**Authors:** A. Mann, P. Soundararajan, S. Shroff

**Affiliations:** Department of Nephrology, Sri Ramachandra Medical College and Research Institute, Porur, Chennai, India

**Keywords:** Human immunodeficiency virus, end stage renal failure, kidney transplantation

## Abstract

Historically HIV positive patients were considered a contraindication for renal transplant. After the year 1996, with the introduction of HAART the retropositive patients live longer and therefore end stage organ disease is now an increasingly important cause of mortality and morbidity in these patients. Here we report our experience for the first time in India. A forty nine year old hypertensive female from Africa who was diagnosed chronic kidney disease and retropositive status, progressed to end stage renal disease and underwent live related renal transplant at our centre.

## Introduction

Historically, HIV positive patients were considered as a contraindication for renal transplant. It was feared that immunosuppressive drugs could also accelerate the progression of HIV. Together with short life expectancy and shortage of organs, the contraindication for transplantation in HIV infected patients was well justified.[1] After 1996, with the introduction of highly active anti-retroviral therapy (HAART), the morbidity and mortality rates have significantly decreased in people infected with HIV.[2] The past experience of solid organ transplantation in HIV infected patients was scarce. It accounted for non-diagnosed patients at the time of transplantation or those who acquired infection after transplantation. Besides, recipients had not received appropriate antiretroviral therapy and some pre-transplantation data, such as CD4 lymphocytes count and viral load, fundamental to know the infection's long-term prognosis, were unknown. And hence, transplantation outcomes were worse than for non-infected patients.[3,4] The change in the natural history of HIV infection in the HAART era, has led us to consider transplantation a therapeutic option in the HIV infected patients with end stage organ disease.[3–5] To date, isolate cases or reduced number of cases of renal transplantation in HIV-infected patients have been reported.[4,6–8] HIV is no longer a contraindication for renal transplant.

## Case Report

A forty nine year old hypertensive female from Africa, a health care worker by profession was detected to be having chronic renal failure (presumed hypertensive nephropathy) in 2005 while she was detected to be HIV positive and was started on HAART (lamivudine, zidovudine and saquinavir). She progressed to end stage renal disease in May 2007 and was started on maintenance hemodialysis and her immunosuppressives were changed to nevirapine, abacavir and lamivudine. In January 2008 with the prospects of undergoing transplant she was investigated. She underwent adequate dialysis, and anemia was managed with iron sucrose and erythropoietin. Her HIV status was found to be non-replicating with HIV RNA levels less than detectable range and she had CD4 counts of 362cells/microlitre. Pre transplant cross match with donor (Brother) was negative. She was worked up as per British Transplant Society[9] guidelines for transplant in HIV positive patient. Vaccines of hepatitis B, hepatitis A and pneumococcal were given to the patient. CMV, EBV serology was found to be negative. No induction therapy was used. As the patient was concurrently using HAART, she was started on cyclosporine, mycophenolate and steroids. She underwent live related renal transplantation on 14 May 2008. She had difficult surgery due to adhesions (past history of caesarian and appendicectomy) and obesity (weight 91 kgs). Postoperatively, the patient had a stormy course complicated by acute tubular necrosis, cyclosporine toxicity, urinary tract infection, suture site infection and delayed graft function requiring dialysis support for four weeks. She also underwent kidney biopsy on 14^th^ postoperative day which was suggestive of cyclosporine toxicity. She was continued on HAART (lamivudine, abacavir and nevirapine modified according to her GFR) and cyclosporine (according to drug levels), mycophenolate mofetil and prednisolone. The patient was finally discharged after six weeks with a stable graft function (*S. creatnine* 2.3 mg/dl) and adequate urine output.

The patient (16 months post transplantation) is asymptomatic and has stable graft function with serum creatinine if 1.1 mg/dl with non replicating HIV status.

## Discussion

Solid organ transplantation was considered as an absolute contraindication to transplantation in pre HAART era. Most of the HIV positive patients who were transplanted in pre HAART era had dismal results.[10–12] These patients in pre HAART were those who were not diagnosed HIV before transplantation due to lack of HIV screening or were those infected by HIV during the transplantation.[10] Poli *et al*. described five cases infected with HIV with median follow up of sixty months.[13] The two studies published in pre HAART era from university of Pittsburg by Tzakes etal found a five year survival of five percent in the patients who were HIV positive before or after the transplantation and most of these patients died of AIDS.[14] With the introduction of HAART in 1996, the HIV associated mortality and morbidity has reduced drastically.[15] The HIV positive patients live longer in HAART era and therefore end stage organ disease is now an increasingly important cause of mortality and morbidity in this group of patients.[16] Because of no published experience in India about kidney transplantation in HIV positive, there are no guidelines available. World experience is also limited about kidney transplantation in HIV positive patients. We decided to follow the guidelines given by British Transplantation Society Standards Committee written on behalf of British HIV association which are available on line.[9] It also gives guidelines regarding use of immunosuppressives in HIV positive patients who are on HAART. The understanding of drug interactions between immunosuppression therapy and HAART is critical to patient management as use of anti retroviral drugs have been associated with significant drug-drug interactions.[17] All protease inhibitors (PI) that exhibit anti HIV activity are metabolized by CYP3A4 isoenzyme.[18] The NNRTI are also extensively metabolized by liver via the same system.[19] Therefore, drug interactions can be anticipated if NNRTI are co administered with other drugs that are metabolized by same metabolic pathway.[20] The protease inhibitors besides being substrates of CYP, also act as inhibitors of P-gp, a transmembrane glycoprotein that functions as an energy dependent efflux pump for a wide variety of structurally unrelated compounds.[21–23] Also, MRP1 and possibly MRP2 are known to be involved in disposition of PI.[22] The calcinurin inhibitor cyclosporine and tacrolimus are also metabolized by liver by CYP3A and cyclosporine is also substrate for P-gp and MRP2 transporters thus, when used concurrently with PI the drug levels of CNI may be increased.[18] MMF also may increase intracellular levels of abacavir, didanosine and tenofovir and result in enhanced toxicity.[18] Therefore, BTS recommends that patients selected for transplant should have a trial of four weeks of CNI and MMF with therapeutic drug monitoring to determine the optimal dose immunosuppressant PIs/NNRTIs on a stable HAART. Once started, regular therapeutic drug monitoring will be required until stable drug levels have been achieved.

There are no recommendations about how to use antirejection therapy in this group of patients. British transplantation society recommends that acute graft rejection should be treated with high doses of injectable methylprednisolone and the treatment of two or more acute rejections is associated with high complication rates. And, therefore it is advised that graft nephrectomy should be considered after two or more episodes of steroid resistant rejection. In view of the complex interaction between different drugs and their unpredictable effect on graft, it is advised that all episodes of graft dysfunction should be assessed with renal transplant biopsy. The use of polyclonal antibodies or OKT3 is not recommended as a rescue therapy for acute rejection.[9]

Besides these medical interactions and unpredictable effects of HAART on kidney transplant and its outcomes, there has been a concern whether transplant benefits the individual patient. With regard to the question of absolute efficacy, transplantation can certainly help HIV positive patients with chronic kidney disease. The question about relative efficacy – all the published reports of transplant in HIV positive patients who are receiving multi drug HAART have concluded that in most cases HIV infection does not affect the outcome of transplantation. There is a concern that therapy might lead progression of HIV. However the experience to date suggests that the use of standard immunosuppressive agents in patients with well controlled HIV infection does not increase their susceptibility to HIV infection or malignant conditions. Also there is concern that intraoperative accidental transmission is falsely hyped. In fact, the risk of transmission is much lower as compared to another infectious disease such as HCV-which is present in many patients who undergo surgery, even if a team member is exposed to HIV various effective post exposure prophylaxis regimes are available.[24]
